# Dynamic regulation of interregional cortical communication by slow brain oscillations during working memory

**DOI:** 10.1038/s41467-019-12057-0

**Published:** 2019-09-18

**Authors:** B. Berger, B. Griesmayr, T. Minarik, A. L. Biel, D. Pinal, A. Sterr, P. Sauseng

**Affiliations:** 1 Department of Psychology, Ludwig-Maximilians-Universität Munich, Leopoldstr. 13, 80802 Munich, Germany; 2 School of Psychology, University of Birmingham, Edgbaston, Birmingham, B15 2TT UK; 3 Fachbereich Psychologie, University of Salzburg, Hellbrunnerstr. 34, 5020 Salzburg, Austria; 4 School of Psychology, University of Minho, Campus de Gualtar, 4810-057 Braga, Portugal; 5 School of Psychology, University of Surrey, Guildford, GU2 7XH UK

**Keywords:** Cognitive neuroscience, Short-term memory, Working memory, Human behaviour

## Abstract

Transiently storing information and mentally manipulating it is known as working memory. These operations are implemented by a distributed, fronto-parietal cognitive control network in the brain. The neural mechanisms controlling interactions within this network are yet to be determined. Here, we show that during a working memory task the brain uses an oscillatory mechanism for regulating access to prefrontal cognitive resources, dynamically controlling interactions between prefrontal cortex and remote neocortical areas. Combining EEG with non-invasive brain stimulation we show that fast rhythmical brain activity at posterior sites are nested into prefrontal slow brain waves. Depending on cognitive demand this high frequency activity is nested into different phases of the slow wave enabling dynamic coupling or de-coupling of the fronto-parietal control network adjusted to cognitive effort. This mechanism constitutes a basic principle of coordinating higher cognitive functions in the human brain.

## Abstract (web summary)

Working memory involves a fronto-parietal brain network, but how the parts of this network are coordinated is unclear. Here, the authors show that fast brain activity at posterior sites is nested into prefrontal slow brain waves, with cognitive demand determining the slow wave phase involved.

## Introduction

When information is processed in working memory (WM)^1^ one can frequently obtain neural activity in dorsolateral prefrontal, anterior cingulate, and posterior parietal cortices comprising a cognitive control network^2–6^. It is suggested that in particular cognitive monitoring functions rely on the interaction between anterior cingulate and posterior parietal cortices^4^. The exact neuronal mechanisms by which posterior brain regions can dynamically access prefrontal resources in situations requiring high demand of cognitive control remain unknown, however.

Allocation of cognitive resources has often been associated with rhythmical fluctuations of electrical brain potentials in a frequency range between 4 and 8 Hz in medial prefrontal cortex (PFC) and anterior cingulate cortex^7–10^—also known as frontal-midline (FM) theta activity. Theta oscillations entrain neuronal spiking as well as fast oscillatory activity in the human and the animal brain^11–17^, and can be found in the neocortex as well as the hippocampus^18^. Fast brain oscillations and neuronal spiking are more likely to occur during the excitatory than the inhibitory phase of the theta period. This mechanism of phase coding has not only been reported on a local scale. Recent evidence in rats and mice suggests that neocortical and tegmental neurons are entrained by prefrontal as well as hippocampal theta oscillations^14,17^—findings well in line with the idea that this kind of temporal coding allows for the precise timing of widespread neuronal activity and thus, supports effective neuronal communication throughout the brain^19^. WM processes particularly engage PFC^20^ and are associated with increased FM-theta activity under increasing load and cognitive demands^21,22^. Hence, we specifically investigated the role of these anterior theta oscillations in the context of the coordination of neuronal activity in remote neocortical areas. We hypothesized that FM-theta activity^23^ and associated phase-based fluctuations of excitability represent a highly efficient gating mechanism^24^ within the fronto-parietal control network allowing or preventing access to prefrontal cognitive resources attuned to cognitive load (difficulty); i.e. constituting a prime mechanism for highly flexible, cost efficient, and dynamic resource allocation. More specifically, we assumed that the relation between FM-theta phase and high-frequency neuronal activity in remote cortical areas would indicate the probability of concurrent neuronal activity within the cognitive control network and, thus, would denote strong coupling or decoupling of this network dependent on cognitive demand. Consequently, task-relevant posterior neuronal activity was expected to occur during time intervals with FM-theta phase being in its excitatory phase when a high level of cognitive control was required. During low cognitive demand a decoupled cognitive control network should be indicated by task-related neuronal activity being associated with the more inhibitory phase of FM-theta activity.

Indeed, here we show that depending on cognitive demand posterior high-frequency activity is nested into different FM-theta phase segments. When task difficulty is high posterior fast activity occurs when FM-theta expresses a trough. When cognitive demand is low it is nested into the peak of FM-theta waves. Effects of transcranial magnetic stimulation to right posterior sites on task performance are modulated in a FM-theta phase-dependent way, suggesting that the here described mechanism of cognitive control is of causal nature.

## Results

### Experiment 1

Electroencephalographic (EEG) signals were recorded from healthy human participants while they performed a visuospatial WM task. In four different experimental conditions one or four spatial target positions in a grid display had to be retained, or mentally mirrored around a vertical gap and then retained. This allowed for a parametric variation of load and cognitive task (LOAD: one vs. four items; CONDITION: retention vs. manipulation).

**Fig. 1:**
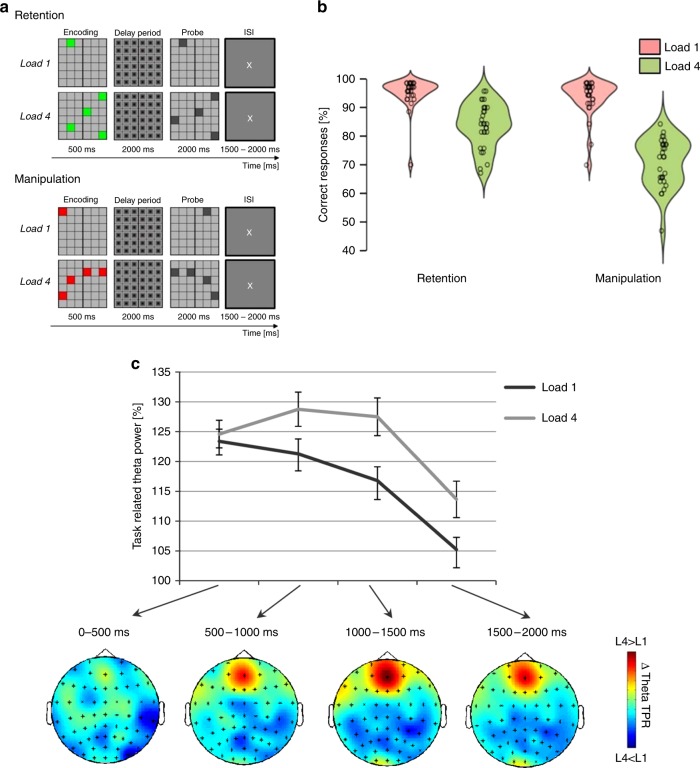
Experimental paradigm, behavioural results and FM-theta amplitude. **a** Schematic depiction of single trials from experiment 1 with conditions ”retention“ and ”manipulation“ and variation in memory load (load 1 vs. load 4). In the retention condition spatial locations of one or four items had to be retained for 2000 ms and then compared to a probe. In the manipulation conditions spatial locations of items had to be mentally mirrored around the vertical gap in the grid, retained in memory and compared to the probe. **b** Accuracy rates (in percent) for the four conditions from experiment 1. Red violin elements represent load 1 conditions, green violin elements indicate load 4. **c** Task-related FM-theta amplitude increase during the delay period at electrode AFz. Values beyond 100% indicate increased amplitude compared to baseline. Error bars represent standard error of mean. Brain maps show the topographical distribution of FM-theta activity difference between load 4 and load 1

*Task accuracy*: Mean accuracy rate (correct responses in percent) was generally higher for retention (89.6 ± 9% SEM) compared to manipulation (82.8 ± 1.1%) and participants generally performed better for load 1 (94.9 ± .7%) compared to load 4 (77.6 ± 1.3%), as indicated by significant main effects for CONDITION (F_1/24_ = 67.63; *p* < .001) and LOAD (F_1/24_ = 280.35, *p* < .001) in an ANOVA. Furthermore, a significant interaction was found for CONDITION × LOAD (F_1/24_ = 38.51, *p* < .001). Post-hoc t-testing (FDR corrected for multiple comparisons;^25^) showed no difference in performance for load 1 between retention (95.7 ± .5%) and manipulation (93.9 ± 1%), whereas for load 4, performance declined by 11.8% for manipulation (71.7 ± 1.5%) compared to retention (83.5 ± 1.5%; Fig. 1b). This pattern of performance results indicates increasing cognitive demand from retention load 1 to manipulation load 4, with a likely ceiling effect for the two load 1 conditions.

*FM-theta Event-related amplitude increase*: In line with previous research indicating that FM-theta amplitude is modulated by cognitive load^7–10^ there were differences between the experimental conditions in FM-theta amplitude: the two more demanding tasks requiring the processing of four targets (retention load 4 and manipulation load 4) elicited significantly stronger FM-theta activity compared to the two easier tasks (retention load 1 and manipulation load 1; main effect for factor LOAD F_1/24_ = 12.4, *p* < .01 in a repeated-measures ANOVA; Fig. 1c), thereby supporting the notion that FM-theta activity is provoked by increased demand on the central executive. In contrast, no main effect of CONDITON was obtained; indicating that the mental operation itself (i.e. maintenance vs manipulation) does not impact on FM-theta activity.

*FM-theta phase to remote gamma amplitude coupling*: Next we investigated how FM-theta phase relates to fast rhythmical brain activity—an indicator of increased neuronal firing and information processing^26–28^—in remote cortical areas using the following methodology: For each single trial and experimental condition, amplitude of EEG activity >30 Hz at any of the recorded EEG sites was sorted according to FM-theta phase and then averaged into equally large theta phase bins. This resulted in an estimate of high-frequency amplitude as a function of FM-theta phase. In a first analysis step all electrode sites at which gamma amplitude was significantly modulated by FM-theta phase (non-uniform distribution of gamma amplitude across the FM-theta cycle indicated by either a significant main effect or significant interaction involving the factor FM-THETA PHASE in repeated-measures ANOVAs; see methods section for details) were identified. At 20 out of 60 electrode sites gamma amplitude was modulated by FM-theta phase. These 20 sites formed three electrode clusters: one frontal cluster (electrode sites FP1, F7, FC5, FC1, FCz, and FC2), one left temporo-parietal cluster (electrode sites C3, T7, CP1, CP3, CP5, CP7, TP7, P5, and P7), and one right temporo-parietal cluster (electrodes C6, CP6, TP8, P2, P4, and P6). For each participant FM-theta phase-sorted gamma amplitude was then averaged across electrode sites for each of the three clusters separately and subsequently cross-correlated with a template in shape of a theta cosine-wave. The same procedure was carried out for single subject data in which FM-theta phase and gamma amplitude data were shifted by one trial. Consequently, in these shifted data sets any relation between FM-theta phase and distant gamma amplitude was spurious. Cross-correlations between the shifted data and a theta cosine-wave were run. Then the absolute maximum from the cross-correlogram based on real and based on shifted data were statistically compared using Wilcoxon-tests. Only for the right temporo-parietal cluster real data showed significantly stronger gamma amplitude modulation by FM-theta phase (as indicated by higher maxima in the cross-correlograms) compared to shifted data (all Z > −2.03, all *p* < .022, FDR-corrected, one-tailed).

**Fig. 2:**
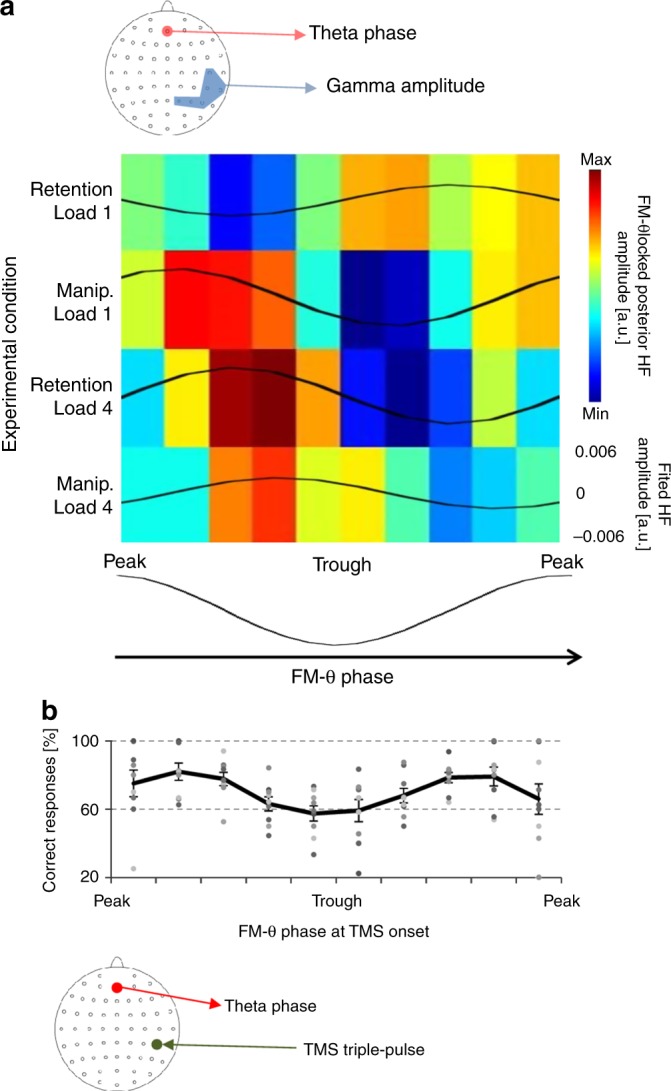
Task-dependent nesting of posterior gamma band activity into FM-theta waves and FM-theta phase-specific TMS effects. **a** FM-theta phase-locked right temporo-parietal gamma amplitude (experiment 1) during the delay period. Mean z-transformed gamma amplitude from electrode sites within the blue-shaded area in the headmap is represented as a function of FM-theta phase extracted from electrode site AFz. Warm colors indicate stronger gamma amplitude. On the *x*-axis ten FM-theta phase bins covering one complete FM-Theta cycle are shown. The four lines of the *y*-axis represent the four experimental conditions ordered according to task difficulty. Note that the more cognitive control an experimental condition required (retention load 1 < manipulation load 1 < retention load 4 < manipulation load 4) the more was posterior gamma activity nested into the through of FM-theta phase. **b** Effect of right temporo-parietal triple-pulse rTMS on task accuracy for manipulation of four items (experiment 2). The strength of the disruptive effect of rTMS depends on instantaneous FM-theta phase at onset of stimulation. When posterior rTMS is applied close to the trough of FM-theta—the sensitive phase into which EEG gamma activity is nested in this condition during experiment 1—mean accuracy rates drop close to chance level. The ten FM-theta phase bins on the *x*-axis are aligned to FM-theta phase from **a**. Dots represent single subject data. The line graph represents sample mean values with standard error of mean as error bars

Interestingly, FM-theta phase associated with maximal right temporo-parietal gamma amplitude differed between conditions: with increasing task difficulty a precession of phase to gamma amplitude coupling towards the trough of the theta cycle was observed (Fig. 2a; results from a repeated-measures ANOVA: significant interaction between LOAD, CONDITION, and FM-THETA PHASE: F_9/216_ = 2.65, *p* = .033, Huynh-Feldt corrected). In the easiest condition (retention load 1) the right temporo-parietal gamma burst was nested shortly prior to the peak of the FM-theta cycle, whereas in the hardest condition (manipulation load 4) this high-frequency activity burst occurred slightly before the trough of the FM-theta period. Thus, the amount of cognitive control required for the task determined the phase of FM-theta to which remote, right posterior gamma amplitude was locked [Note that there were no overall (independent of FM-theta phase) differences of gamma amplitude between tasks. A 2^∗^2 ANOVA with factors LOAD (1 vs 4) and CONDITION (retention vs manipulation) on event-related amplitude increase yielded no significant main effect or interaction (all Fs_1/24_ < 1.6, all ps > .21).]. This suggests a dynamic and effort-related modulation of the interaction between prefrontal and temporo-parietal regions through fine-grained alterations in the phase-coupling between nested frequencies. More specifically, the pattern of FM-theta phase to high-frequency amplitude coupling suggests that FM-theta phase provides time windows during which access to prefrontal cognitive resources is allowed and denied in a predictable and cyclic fashion in order to be reliably available if distant task-relevant processing is aligned accordingly. In other words, FM-theta oscillations allow for precise alignment of processing achieving optimal conditions for inter-regional communication, while taking into account the current task requirements. Hence, FM-theta very much has the role of a gatekeeper, and this mechanism acts as a highly efficient gating mechanism for the allocation of and access to cognitive resources.

It is important to emphasize that no significant local theta phase to gamma amplitude coupling was obtained. That means that neither for the electrode site expressing strong FM-theta was there any significant modulation of local gamma amplitude by theta phase, nor was there any significant modulation of temporo-parietal gamma amplitude by temporo-parietal theta phase (for each condition and site main effect for factor PHASE in a repeated-measures ANOVA: all Fs_9/216_ < 1.63, *ps* > .180; moreover, there were no significant other main effects or interactions for local theta phase to gamma amplitude coupling either: all Fs_9/216_ < 1.10, *ps* > .372.).

Interregional within-frequency phase coherence between frontal-midline and right temporo-parietal sites was evaluated for theta and for gamma frequency bands separately using paired-sample *t*-tests. Compared with a pre-stimulus baseline interval there was only a significant increase of theta phase coherence during the first 500 ms of the delay interval for the retention of four items between electrode sites AFz and P2 (*p* < .05). No significant increase in phase coherence was obtained at any other time interval, for any other experimental condition, and any further electrode pair at theta or gamma frequency. Thus, out of 192 comparisons only one (or .052%) was significant on the uncorrected 5% significance level. We could not find any evidence for increased coherence between frontal-midline and right temporo-parietal electrodes during the delay period.

Given that right temporo-parietal gamma amplitude was associated with different FM-theta phases depending on task difficulty, we predicted that the impact of disrupting fast rhythmical brain activity within this temporo-parietal brain region with transcranial magnetic stimulation (TMS) should depend on the timing of TMS pulses with respect to the ongoing FM-theta phase (see experiment 2).

### Experiment 2

To demonstrate a causal relationship between the above described nesting of posterior gamma bursts into specific FM-theta phases and task performance, we ran an experiment in which FM-theta phase-dependent neurostimulation was applied. EEG was recorded while participants performed the most difficult condition from experiment 1 (manipulation load 4; see above). In each single trial, randomly within the delay interval, a fast triple-pulse of TMS was delivered to the task-active right temporo-parietal site. Offline, the instantaneous phase of FM-theta was obtained for the time point when TMS was applied over the right posterior cortex, and single-trial task performance was sorted accordingly. This resulted in an estimate of task performance as a function of instantaneous FM-theta phase at which right temporo-parietal TMS had been applied. The idea behind this was that, as suggested by experiment 1, task-active posterior neuronal firing was synchronised to FM-theta phase, and this communication pattern serves as a gating mechanism to prefrontal cognitive resources. Consequently, TMS should be differentially effective in disrupting task performance dependent on instantaneous FM-theta phase: If (incidentally) TMS at posterior sites is delivered during the trough of the FM-theta cycle (the preferred phase for neural activity of task-active posterior neurons in this condition, as suggested by experiment 1) efficient neuronal processing should be disrupted. If, however, TMS pulses are applied while FM-theta expresses a peak, i.e. the un-preferred phase of the FM-theta wave, magnetic stimulation should be inefficient in disrupting task performance.

Nicely in line with the pattern we found in the initial EEG data (Fig. 2a), task performance was significantly modulated by TMS depending on FM-theta phase during stimulation onset (repeated-measures ANOVA: F_9/72_ = 2.73, *p* = .008, η² = .26, BF_10_ = 8.02). Importantly, right temporo-parietal TMS applied during the peak of the FM-theta cycle virtually did not impact on task performance at all. However, if applied shortly before the trough of FM-theta—the phase to which increased gamma amplitude had been locked in experiment 1—right posterior TMS led to a drop in performance close to chance level (Fig. 2b). This directly suggests that task-relevant right temporo-parietal neural activity is pulsed and synchronised to FM-theta waves and further led to the proposition that the trough of FM-theta represents the phase during which frontal cognitive processing is most active and accessible to distant regions executing task-relevant processes that align to it. By contrast, the peak seems to be the phase that isolates frontal resources from distant regions.

### Experiments 3a and 3b (control experiments)

In two control experiments we investigated the specificity of the results from experiment 2 (combined EEG and TMS experiment). In control experiment 3a we tested whether TMS delivered over a (task irrelevant) control site showed a comparable effect as reported in experiment 2. TMS over right posterior parietal sites could also have had unspecific effects, such as stimulation of peripheral nerves and muscles in the scalp and auditory stimulation due to clicking of the coil. Thus, it could be argued that this sensory stimulation would interfere in a FM-theta phase-dependent way with task performance, completely independent of actual stimulation of right posterior parietal cortex. To rule that interpretation out, we ran an experiment in which we delivered TMS over the vertex (instead of right posterior parietal sites). Otherwise control experiment 3a was identical to experiment 2 above. An ANOVA comparing percentage of correct responses in the working memory task across ten FM-theta phase bins at which TMS had been delivered (as in experiment 2) did not indicate any significant effect (repeated-measures ANOVA: F_9/81_ = 1.50, *p* = .161, η² = .14, BF_01_ = 2.41; see Supplementary Fig. 2a). Therefore, we can conclude that FM-theta phase-dependent interference of working memory performance by TMS seems specific for right temporo-parietal stimulation sites.

In experiment 1, retention of only one spatial position in working memory led to right posterior parietal gamma activity being nested into the peak of FM-theta; whereas mental manipulation of four items resulted in right posterior parietal gamma waves locked to the trough of FM-theta. Consequently, the effect described in experiment 2—disruption of task performance when posterior parietal TMS pulses are delivered at FM-theta trough—should be specific for mental manipulation of four spatial positions. If retention of one item can be disrupted by right posterior parietal TMS at all, then this should only be possible with TMS being delivered at FM-theta peaks. Therefore, in control experiment 3b an identical setup as in experiment 2 was used with the exception that participants carried out the easiest of the four working memory conditions: the retention of only one spatial position in each trial. The percentage of correct responses in this task was not dependent on instantaneous FM-theta phase at which posterior parietal TMS was delivered (repeated-measures ANOVA comparing percentage of correct responses across ten FM-theta phase bins: F_9/81_ = 0.92, *p* = .513, η² = .10, BF_01_ = 8.08; see Supplementary Fig. 2b). This indicates that a phase-specific interference at the FM-theta trough is not an unspecific effect but specific for the most difficult experimental condition deploying the most frontal resources, i.e. the condition that also shows posterior gamma activity locked to the FM-theta trough. However, it should be noted that most participants performed at or very close to ceiling in the easiest experimental condition (retention of one item); average score of correct responses was at 97.0% (std = 7.89). This could also explain why TMS delivered at the FM-theta peak (preferred phase for the easiest condition) did not impact on task performance.

### Experiment 4 (internal replication and direct comparison of effect and control)

In experiments 2 and 3 the tested sample sizes were small. Nevertheless, Bayes factors indicated substantial evidence for the alternative hypothesis in experiment 2 and for the null hypothesis in experiment 3b. A problem, however, is that the effects from experiment 2 and the control experiments cannot be directly compared in a within-subject design. Therefore, an experiment in which each participant underwent right parietal TMS (identical to experiment 2) and, in a separate session, with each participant being stimulated over a control site, was carried out. This experiment was designed as an internal replication attempt of the effect from experiment 2; and it allowed direct statistical comparison between effects, and aimed to better control for TMS-non-specific spurious effects (it has been discussed that TMS over the vertex as used in experiment 3a does not control very well for muscle twitches and cutaneous sensation^29,30^). Each participant performed exactly the same task with exactly the same TMS protocol as in experiment 2 in one session; in the other session TMS was applied over a right fronto-central electrode position that can be considered as a control stimulation site with at least as much cutaneous and muscle stimulation as the right parietal target site^29^. A two-way repeated-measures ANOVA with factors STIMULATION SITE and THETA PHASE showed a significant interaction effect (F_9/99_ = 2.31, *p* = .021, η² = .17, BF_10_ = 1.48; one-tailed post-hoc *t*-tests indicate significantly reduced task accuracy when TMS was delivered over electrode site CP6 compared to FC6 just before the trough of FM-theta phase, i.e. in phase bin 4 (t_12_ = 3.00, *p* = .006, BF_10_ = 10.58) and phase bin 5 (t_12_ = 4.29, *p* < .001, BF_10_ = 65.08); no other phase bins (except the ones prior to the FM-theta trough) exhibited a significant difference between TMS sites; for a depiction of results see Supplementary Fig. 3a). To compare effects from experiment 4 with those from experiments 2 and 3, one-way repeated-measures ANOVAs with factor THETA PHASE were run separately for right parietal stimulation site and right fronto-central TMS. Findings from experiment 2 were successfully replicated with right parietal TMS (F_8.6/112.4_ = 2.17, *p* = .034, Huynh-Feldt-corrected, η² = .14, BF_10_ = 1.65). However, note that effect size in the replication attempt was clearly smaller than in experiment 2 (η² = .14 vs. η² = .26). A reduction in effect size might be explained by the fact that, in general, participants in experiment 4 were doing much better than those in experiment 2. Some of the participants in experiment 4 were performing close to ceiling level, which could lead to less performance modulation via TMS. In the control condition, right fronto-central TMS did not impact significantly on task-performance in a FM-theta phase-specific way (F_9/108_ = 1.36, *p* = .216, η² = .10, BF_01_ = 4.64). Finally, data from experiment 2 and the right parietal TMS session from experiment 4 were put together and analysed with a one-way repeated-measures ANOVA. The main effect for factor THETA PHASE was significant (F_7.6/175.6_ = 3.10, *p* = .003, Huynh-Feldt-corrected, η² = .12, BF_10_ = 13.63; see Supplementary Fig. 3b).

## Discussion

The current study strongly suggests that the phase of FM-theta provides time windows in which distributed neuronal activity can be synchronised during higher cognitive processes that require inter-regional co-operation. It is demonstrated that right temporo-parietal high-frequency EEG activity is nested into FM-theta waves. Importantly, the alignment of this nesting with the theta peak or theta trough is dependent on the cognitive control required to effectively perform a working memory task. Specifically, our study shows that under low load conditions posterior gamma activity is aligned towards the frontal theta peak while under high load conditions it is aligned to the frontal theta trough. Moreover, we suggest that this long-range synchronisation of task-relevant neuronal processing goes along with rhythmic fluctuations of frontal neural excitability, allowing flexible access to/distribution of frontal cognitive resources adjusted to the current task requirements. Critically, our experiments demonstrate that the relationship between cognitive control required and this theta-gamma alignment is causal: TMS delivered over the right temporo-parietal cortex only disrupted WM task performance when applied while FM-theta was at its excitatory phase (i.e. near the trough) and thus at the phase to which temporo-parietal gamma activity was coupled in the respective experimental condition, highlighting the direct causal relevance of this mechanism. This also suggests that fronto-parietal interaction relevant for WM control functions only takes place in short, periodic time windows during which communication between distant regions is either facilitated or disrupted depending on task demands.

**Fig. 3:**
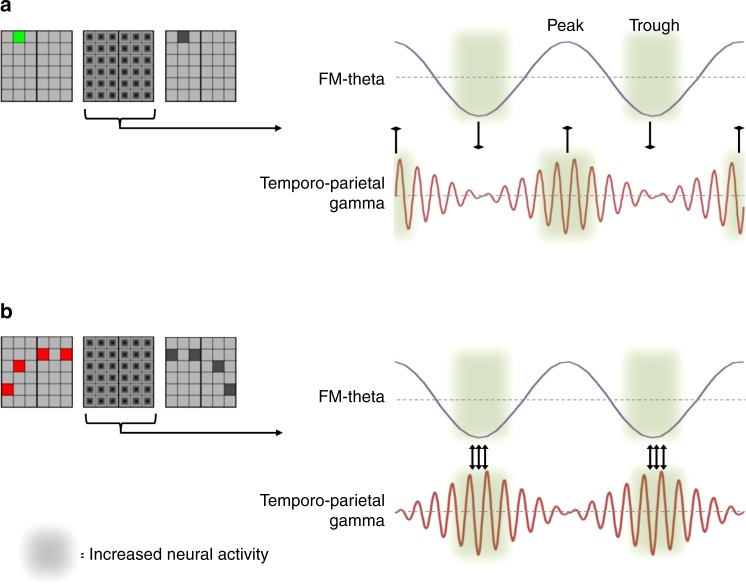
Dynamic coupling and decoupling of fronto-parietal brain networks by phase-dependent nesting of gamma band activity into FM-theta waves. Schematic depiction of alignment of frontal and posterior neuronal activity by theta-gamma phase-amplitude coupling. **a** A fairly easy working memory process will not require deployment of large amounts of cognitive resources. Temporo-parietal cortex does not need to be coupled to fronto-medial cortex. Neuronal activity in frontal cortex is paced by FM-theta phase, with increased neuronal firing at the trough compared to the peak of the theta wave. Gamma activity is a signature of strong neuronal firing in temporo-parietal cortex. If posterior gamma activity is, thus, nested into the peak (the inhibitory phase) of FM-theta there will be un-simultaneous neural firing and therefore a state of decoupling within the fronto-parietal network. **b** A challenging working memory task such as the mental manipulation of four items will require maximal allocation of cognitive resources. Temporo-parietal cortex will need access to prefrontal cortex. This is achieved by alignment of frontal and posterior neural firing, enabled by nesting of posterior gamma activity into the excitatory trough of FM-theta phase. This will lead to effective coupling within the fronto-parietal network, with a dynamic adjustment of this neural synchronisation pattern dependent on cognitive resource allocation to the particular task

Together, the current findings indicate that the relative alignment of posterior gamma to the FM-theta peak or trough represents a highly efficient gating mechanism, which controls access to/distribution of frontal cognitive resources through the dynamic synchronisation or desynchronisation of fronto-posterior networks. Similarly, we demonstrated that this active mechanism of decoupling can also be found in the default-mode network while healthy young participants are engaged in a demanding working memory task^31^. This theory is further supported by studies indicating that local neural activity is modulated by slow oscillations^10,32,33^. Specifically, Haegens et al.^34^ reported the trough of slow oscillatory activity being associated with higher neuronal spiking than the more inhibitory peak of slow brain waves, a principle also suggested to hold true for theta activity^10,33^. Hanslmayr and colleagues^35^ were able to show in humans that visual perceptual performance was enhanced when stimuli were presented around the trough of a theta wave. Moreover, this was associated with stronger neural activity in posterior brain areas and increased functional coupling between occipital and parietal cortex. A recent study by Voytek et al.^36^ even found frontal theta phase modulating local gamma activity in intracranial recordings from epileptic patients. Recently, Alekseichuk et al.^37^ delivered cross-frequency transcranial alternating current stimulation over the prefrontal cortex; they found that high-frequency gamma stimulation in combination with the peak of a theta stimulation (inducing gamma activity locked to the frontal theta trough on cortical level) increased working memory performance and long-range connectivity in the brain. Based on this evidence across species and across methods, we theorize that PFC exerts greater neuronal activity during the theta trough than the theta peak. Moreover, oscillatory brain activity in the gamma range has been proposed as a marker of increased neural firing^26^. At the same time it is well accepted that right temporo-parietal brain areas are associated with the processing and storage of visuospatial information^38,39^. Consequently, when—like in the easiest experimental condition in the current study (retention load 1)—posterior gamma activity is coupled to the peak of FM-theta this suggests that task-relevant neural activity in right temporo-parietal cortex and large parts of this fronto-medial brain region are not occurring simultaneously. Given that increased neuronal activity in right temporo-parietal cortex (as indicated by gamma activity) and the excitatory FM-theta phase are separated by up to 100 ms it is very unlikely that much neural communication between fronto-medial and temporo-parietal cortex takes place in this condition (Fig. 3a). One could argue that an easier mechanism of decoupling would simply be having no phase synchronisation of oscillatory activity between frontal and posterior brain areas at all. However, if these distant brain regions are tightly (indirectly) connected, even an increase of unspecific noise might lead to spurious and therefore interfering coupling between anterior and posterior brain areas. By actively nesting posterior neural activity into the inhibitory frontal theta phase spontaneous and potentially interfering fronto-parietal coupling becomes less likely. The retention load 1 condition is easy enough to allow effective task performance without draining cognitive resources. We therefore argue that fronto-medial cortical regions associated with cognitive control and monitoring processes seem to actively desynchronise with the temporo-parietal cortex by non-simultaneous neuronal firing in situations where little cognitive resources are required. In situations where more cognitive resources have to be deployed to ensure effective performance, such as greater task difficulty or task complexity, fronto-parietal coupling is actively facilitated through the alignment of temporo-parietal gamma bursts with the excitatory phase of theta. With time windows of increased neural activity (FM-theta trough and posterior gamma bursts) in frontal monitoring areas and posterior visuospatial regions more and more overlapping in this way, it is suggested that there is increasingly simultaneous neuronal firing between right temporo-parietal and fronto-medial cortex (Fig. 3b). Thus, the described mechanism of theta oscillations organizing time-windows for optimal synchronisation between frontal and posterior brain activity seems very efficient in gating access to/distribution of cognitive resources governed by fronto-medial brain areas to remote task-relevant regions.

Further support for the gating mechanism outlined above comes from a study by Polanía et al.^40^ who were able to increase working memory performance by applying transcranial alternating current stimulation (tACS) at theta frequency over frontal and parietal cortex simultaneously. Importantly, performance was only increased when stimulation was applied at zero phase lag between frontal and parietal cortex. When stimulation with an offset of 180 degrees was applied (excitatory FM-theta trough occurring simultaneously with inhibitory parietal theta peak), working memory performance was impaired. This is in good agreement with our current results. Synchronous theta stimulation, i.e. with zero phase-lag (excitatory FM-theta trough reoccurring with excitatory parietal theta trough), would allow simultaneous neural activity in frontal and parietal cortex. Phase-reversed neurostimulation at theta frequency, i.e. where the tACS induced excitability fluctuations are phase shifted by 180 degrees between frontal and parietal cortical regions, very similar to when posterior gamma activity is aligned to the inhibitory frontal theta phase, might lead to non-simultaneous neural activity in a fronto-parietal network and consequently to a communication breakdown between the involved brain regions.

In a recent study by Daume et al.^41^, an alternative mechanism by which FM-theta might control fast oscillatory activity in posterior brain regions during visual working memory has been suggested. FM-theta was found to be top-down coupled to theta activity in inferior temporal cortex. In inferior temporal cortex, on the other hand, local coupling between theta phase and gamma amplitude was observed. In our data, in contrast, we found neither increased inter-regional coherence at theta frequency between medial frontal and posterior sites, nor did we obtain local coupling between theta phase and gamma amplitude. Instead, the current findings indicate that FM-theta defines brief time intervals (around the trough of the theta wave) in which prefrontal cortex is susceptible for neural communication (based on a general mechanism as described in the communication through coherence framework^42^). Demanding cognitive processing will rely on higher sensory areas having access to prefrontal cortex. Consequently, neurons in sensory areas will have to be activated during time windows in which the prefrontal cortex allows access and neurons are most excitable. No long-range theta coherence would be required for that, and theta oscillations would not carry any information per se. They would just be involved in defining time windows to which neuronal firing can be synchronised, resulting in or preventing fairly simultaneous firing of remote neurons. In conditions that do not require cognitive control (e.g. highly trained or very easy cognitive processes) sensory neurons would be “denied access” to the prefrontal cortex by having them activated whenever there is low susceptibility for communication in the prefrontal cortex (i.e. around the FM-theta peak). This would be a more effective mechanism of actively decoupling inter-regional networks than simply reducing coherence. By virtue of this process while a particular brain region remains decoupled from frontal cortex, other cortical areas might be granted access, and indeed, Pinal et al.^31^ were able to show that in a visual WM task gamma activity from right temporo-parietal cortex (i.e. task-relevant) and from precuneus (i.e. default-mode network) were associated with frontal-midline activity that was exactly phase reversed: In healthy, young participants task-relevant right temporo-parietal gamma was nested into the trough, whereas default-mode network associated gamma activity from the precuneus was associated with the peak of frontal-midline oscillations. This makes it appear likely that although right temporo-parietal cortex is decoupled from prefrontal cortex in the easy conditions in the present study, other cortical sites might be nesting their gamma activity into the trough of FM-theta activity. In the easy tasks posterior brain areas can perform without additional support from prefrontal cortex; participants might even think of something completely task-irrelevant while they can still do the task well, or they might show increased activity in a default-mode network. Cortical areas involved in task-irrelevant processing or default-mode activity might then be the ones coupled to prefrontal cortex (however, since this activity would not be coherent across participants it would not show up in sample statistics). Thus, we would speculate that the here reported mechanism of controlling long-range interactions in the brain with specific coupling and decoupling going on in parallel could be a general process how cognitive control is implemented in the brain.

The current findings present converging evidence for FM-theta activity playing a key role in a neuronal mechanism by which fronto-parietal synchronisation, and thus distribution of/access to prefrontal cognitive resources to/by parietal cortex, is regulated—well in line with recent research in monkeys^43^. Moreover, the current findings underpin the importance of brain oscillatory phase in the coordination of neuronal firing—not merely on a local but even on an inter-regional scale. Could, therefore, the described mechanism be a general principle of how the brain coordinates parallel processes and dynamically controls the allocation of resources towards cognitive tasks? We would argue that at least for processes involving the medial PFC this would be the case, and in fact, there is a broad spectrum of cognitive processes all associated with FM-theta activity generated in the medial PFC^8^, in particular tasks involving parallel processing and the precise control of cognitive resources. Whether the suggested neuronal mechanism also exists for other brain frequencies and structures, however, needs to be addressed by further investigation.

## Methods

### Experiment 1

*Participants*: EEG was recorded from 30 volunteers after giving written informed consent. Five subjects were excluded from analyses due to too many blinks and horizontal eye movements. The mean age of the remaining sample (*n* = 25) was 24.8 years (SD = 3.1). Sixteen out of 25 subjects were male, nine were female. All subjects had normal or corrected to normal vision and were not affected by neurological or psychiatric disorders. Handedness was assessed according to the Edinburgh-Handedness-Scale^44^ which detected 22 right handed and three left-handed subjects. Subjects were financially compensated for participation. The study was approved by the University of Surrey Ethics Committee.

*Experimental design*: Participants performed a visuospatial delayed match to sample task (see Fig. 1a) in a dimly lit room. Stimulus material was presented on a 24” LCD monitor with Presentation® 0.71. At the beginning of each trial a 6 × 6 matrix (covering a visual angle of 6.2° × 6.2°) was presented for 500 ms containing either one or four colored squares (load 1 vs. load 4). If the squares were shown in green, participants had to maintain their positions in memory for 2000 ms (retention condition). If they were shown in red, participants had to mirror their positions around a vertical gap in the matrix and keep the new positions in memory (manipulation condition). Then, a probe matrix with one or four grey labelled squares, depending on the number of previously presented squares, was presented for 2000 ms. Subjects had to indicate by button press whether the maintained/mirrored positions matched the squares in the probe matrix or not. During the inter-trial interval which was jittered with a duration between 1500 and 2000 ms, a central fixation cross was presented. For each task, manipulation load 1 (ManL1), manipulation load 4 (ManL4), retention load 1 (RetL1), retention load 4 (RetL4) 70 trials were run. Half of the trials were match and the other half non-match trials. In each non-match trial, the position of one square changed location by one square (i.e. one square location further left, right, up, or down from the original location). Trials with different tasks were presented in randomized order and luminance was equal between red and green squares. In order to avoid afterimages during the delay interval the matrix shown during the delay period was filled with a grey/black pattern. Participants were instructed to answer as correctly as possible. A training block was carried out at the beginning of the experiment.

Behavioural data were (normally distributed and) statistically analyzed using a two-way repeated-measures ANOVA with factors CONDITION (retention vs. manipulation) and LOAD (load 1 vs load 4). Mean accuracy rate was the dependent variable. Post-hoc *t*-tests were corrected for multiple comparison using Benjamini and Hochberg’s false discovery rate (FDR) correction method^25^.

*EEG data acquisition*: EEG was recorded from 60 scalp electrodes (Ag/AgC1 ring electrodes; Easycap®) mounted according to the international 10-10 system against a nose reference. Signals were amplified with a 64-channel amplifier system (BrainAmp, Brain Products®). In order to control for vertical and horizontal eye movements two electrodes were placed superior to and next to the right eye. Two additional electrodes were mounted on the left and right earlobe for re-referencing the data offline to a digitally linked earlobe reference. The ground electrode was set at the fore-head. EEG-signals were registered between 0.016 and 80 Hz with a sampling rate of 1000 Hz. A Notch-Filter was set at 50 Hz and impedances were kept below 20 kΩ.

*EEG data analyses*: EEG data were analyzed by using BrainVision Analyzer 2.0 (Brain Products®) and Matlab 7.9.0.529 (The Math Works, Inc., Natick, Ma, USA). Statistical analyses were carried out with SPSS, JASP, and Matlab. Data were offline re-referenced to digitally linked ear-lobes and high-pass filtered with a low cutoff at 1 Hz, 48db/Oct (Butterworth Zero Phase IIR Filter, as implemented in BrainVision Analyzer 2.0). ICA ocular correction was applied in order to remove eye blinks and eye movements. Data were then inspected manually and corrected for remaining artefacts. Afterwards, Laplacian current source density (CSD) was calculated in order to attenuate micro-saccadic eye movements leading to spurious power effects and phase synchrony;^45,46^ and to attenuate effects of volume conduction on inter-regional phase synchronisation. For data analyses, data were segmented into epochs of 3100 ms for each experimental task separately (comprising a 600 ms baseline period, a 500 ms encoding matrix and a delay period of 2000 ms). All artefact-free trials, involving correct as well as incorrect responses, were used. The mean number of artefact-free trials for retention load 1 and load 4 was 57.5 (SD = 7.9) and 59.4 (SD = 7.03), respectively. For manipulation the mean number of artefact-free trials was 58.5 (SD = 7) for load 1 and 59.1 (SD = 6.3) for load 4. The analyses as described below were applied to the 2000 ms delay period. For statistical analyses, the delay period was divided into 4 time windows (time 1: 500–1000 ms, time 2: 1000–1500 ms, time 3: 1500–2000 ms, and time 4: 2000–2500 ms).

Analyses of event-related amplitude (ERA) increase/decrease: For ERA calculation, single trials were first submitted to complex Morlet wavelet filtering (as implemented in BrainVision Analyzer 2.0). Data were filtered separately for low (1–30 Hz) and high (30–70 Hz) frequencies in 1 and 10 Hz steps, respectively (continuous wavelet demodulation using a eight cycle Complex Morlet for low frequencies and a 10 cycle Complex Morlet for high frequencies). Amplitude estimates of filtered trials were then averaged for each condition and ERA was calculated. ERA is defined as the percentage of increase or decrease with respect to a predefined baseline period ([activity period—baseline period]/baseline period × 100). We extracted a baseline period from −500 to −200 ms for low frequencies and from −300 to −200 ms for high frequencies prior to the encoding matrix. Amplitude estimates from the delay interval were then averaged into four time windows (as described above). For statistical analysis a three way ANOVA with factors CONDITION (retention, manipulation), LOAD (load 1, load 4), and TIME (time 1, time 2, time 3, time 4) was run for theta (4–7 Hz) ERA at electrode AFz. We selected AFz for statistical analysis, as we found strongest synchronisation at this site compared to neighbouring electrodes. Data were normally distributed.

Frontal-midline Theta locked Gamma Amplitude Modulation: For each experimental task, single-trial phase values for theta (5 Hz) at AFz and single-trial amplitude values for gamma at 30, 40, 50, 60, and 70 Hz (10 Hz frequency bins) at 60 electrode sites were estimated (for wavelet filter parameters see above). Then, z-transformed amplitude values of 30, 40, 50, 60, and 70 Hz were sorted with respect to instantaneous AFz theta phase angles (see ref. ^16^ for a similar approach) and averaged into 10 theta phase bins (each bin covering 36° of a theta cycle). Data were normally distributed. As an explorative analysis, five way repeated measure ANOVAs with factors FREQUENCY (30, 40, 50, 60, and 70 Hz), TIME (time 1, time 2, time 3, time 4), CONDITION (retention, manipulation), LOAD (Load 1, Load 4), and FM-THETA PHASE (segment 1–10) were calculated for each of the 60 electrodes with FM-theta phase-sorted gamma amplitude as the dependent variable. This was done in order to determine whether there is a significant main effect or any interaction involving the factor FM-THETA PHASE as an indicator for modulation of gamma amplitude by FM-theta phase. Explorative analysis revealed three electrode clusters showing gamma amplitude modulation by FM-theta phase: a frontal cluster (FP1, F7, FC5, FC1, FCz, and FC2), a left temporo-parietal cluster (C3, CP1, CP3, CP5 T7, TP7, P7, and P5), and a right temporo-parietal cluster (C6, CP6, TP8, P2, P3, and P6). In a next step, analyses were focused onto these three clusters and five way repeated-measures ANOVAs with factors FREQUENCY, TIME, CONDITION, LOAD, and FM-THETA PHASE with frontal-midline theta phase-sorted gamma amplitude averaged within each of the three electrode clusters as the dependent variable were applied.

Theta Phase and Gamma Amplitude Cross-Correlations: In order to further evaluate whether gamma amplitude is significantly modulated by FM-theta phase, the cosine function of a theta cycle at a frequency of 5 Hz (amplitude ranging from +1 to −1 µV and sampled at 50 Hz, i.e. the same temporal resolution as the FM-theta phase-sorted gamma amplitude averages) was cross-correlated with the theta phase-sorted gamma amplitude for each task within an electrode cluster (see above), separately. Two full theta periods (and equivalent sorted gamma amplitudes) were used for cross-correlations (resulting in 20 data points for each time series). If posterior gamma amplitude was not modulated by FM-theta phase one would expect a flat cross-correlogram with coefficients close to zero. Systematic gamma amplitude modulation by FM-theta phase, however, should result in a cross-correlogram with a clear peak different from zero. This cross-correlation approach was applied to each participant separately; the absolute maximum of the cross-correlogram was then used as a measure for theta-phase modulation of gamma amplitude. Next, in each participant single-trial FM-theta phase and gamma amplitude from the previously identified electrode clusters were shifted by one trial. Consequently, there should not have been strong association between FM-theta and remote gamma amplitude in these shifted data anymore. The cross-correlation approach was repeated for the shifted data. Absolute maxima from the cross-correlogram were then statistically compared to those from the real data after Fisher-Z transformation of the data. Even after Fisher-Z transformation data were not normally distributed. Thus, one-tailed Wilcoxon tests were used to compare crosss-correlation coefficients from real versus shifted data for each task condition and electrode cluster separately. Results were FDR-corrected for multiple comparisons.

### Experiment 2

*Participants*: Ten healthy volunteers (six females; mean age 25.7, (SD = 3.7)) were tested after giving written informed consent. All but one of the participants were right handed, and none of the volunteers suffered from any psychiatric or neurological disorders. Participants had normal or corrected to normal vision. The study was approved by the University of Surrey Ethics Committee.

*Experimental design*: A similar experimental design as in experiment 1 was used. The exception was that only trials with four items that had to be mentally manipulated were used (manipulation load 4). The experiment consisted of a total of 280 trials. In each of the trials the memory set was shown for 500 ms, followed by a 2000 ms delay period and 2000 ms of presentation of the probe stimulus and a variable inter-trial-interval between 1500 and 2000 ms (see experiment 1 for details). In this experiment, however, in 210 trials a TMS triple-pulse was delivered within the delay period. The onset of the triple-pulse was jittered between 500 and 1500 ms after memory set offset. Subjects were instructed to answer as correctly as possible. A training block was carried out at the beginning of the experiment.

*EEG data acquisition*: EEG data were recorded from 30 scalp sites arranged according to the extended 10-10-system using a BrainAmp MRplus amplifier (BrainProducts®) and a TMS compatible electrode cap with Ag–AgCl electrodes (Easycap®). Signal was acquired at a rate of 1000 Hz in a frequency range between 0.016 and 80 Hz (with a notch filter at 50 Hz). During recording a reference on the tip of the nose was used and the ground electrode was placed at electrode position FPz. Horizontal and vertical EOG was recorded.

*TMS protocol*: A MagStim Rapid2 TMS stimulator (MagStim®) with a 7 cm figure-eight coil (MagStim®) was used. In TMS trials a 50 Hz triple-pulse at 80% of individual resting motor threshold was delivered to the right parietal cortex. The stimulation site was EEG electrode site CP6, a position directly in the middle of the right posterior electrode cluster identified in experiment 1. Mean stimulation intensity was 43.7 (SD = 5.4)% maximal stimulator output. The triple-pulse was delivered during the delay period with an onset jittered across trials (see above). EEG was recorded throughout the experiment.

*EEG data analysis*: Preprocessing and artefact rejection was done as described for experiment 1. Data were segmented into epochs starting 1000 ms prior to and ending at onset of the TMS triple-pulse. For electrode site AFz segments were then filtered between 4 and 7 Hz, and instantaneous phase was derived. EEG phase at TMS onset was estimated based on the value gathered 91 ms prior to the triple-pulse (half period of the centre frequency at 5.5 Hz) to avoid results being influenced by filter ringing artefacts at the segment edges (see SOM results from experiment 4 on the reliability of this phase estimation). Trials were then sorted into ten FM-theta phase bins of 36˚ and the rate of correct responses was calculated for each of the theta phase bins. One participant performed more than 2.5 standard deviations below the sample’s mean accuracy (and therefore clearly performed on chance level) had to be excluded from statistical analysis as an outlier. One-way repeated-measures ANOVAs with factor THETA PHASE comparing accuracy rates across the ten FM-theta phase bins was run. To obtain Bayes factors a JZS Bayes factor ANOVA with default prior scales was calculated in JASP.

### Experiment 3

Control experiments 3a and b.

*Participants*: Ten healthy volunteers (five females; mean age 25.3, (SD = 7.5)) were tested for experiment 3a; and eleven healthy volunteers (seven females; mean age 27.33, (SD = 6.6)) completed experiment 3b after giving written informed consent. All of the participants were right handed, and none of the volunteers suffered from any psychiatric or neurological disorders. Participants had normal or corrected to normal vision. The experiment was approved by the local ethics committee.

*Experimental design*: A similar experimental design as in experiment 2 was used for both control experiments 3a and b. For experiment 3a the difference was that in order to establish whether the FM-theta phase-dependent performance impairment was due to unspecific TMS effects (e.g. clicking noise) or to the parieto-temporal stimulation itself, we stimulated over a control site that should not be involved in the task, i.e. the vertex. In experiment 3b the aim was to investigate whether the retention of one item (where posterior gamma bursts were locked to the peak of FM-theta) can be similarly disrupted with TMS. Hence, participants carried out the easiest task (retention load 1) of experiment 1 only. The experiments consisted of 280 trials each. In each of the trials the memory set was shown for 500 ms, followed by a 2000 ms delay period and 2000 ms of presentation of the probe stimulus and a variable inter-trial-interval between 1500 and 2000 ms (see experiment 2 for details). In 210 trials of each experiment a TMS triple-pulse was delivered within the delay period. The onset of the triple-pulse was jittered between 500 and 1500 ms after memory set offset. Subjects were instructed to answer as correctly as possible. A training block was carried out at the beginning of the experiment.

*EEG data acquisition*: EEG data were recorded from 30 scalp sites arranged according to the extended 10-10-system using a BrainAmp MRplus amplifier (BrainProducts®) and a TMS compatible electrode cap with Ag-AgCl electrodes (Easycap®). Signal was acquired at a rate of 1000 Hz in a frequency range between 0.016 and 80 Hz (with a notch filter at 50 Hz). During recording a reference on the tip of the nose was used and the ground electrode was placed at electrode position FPz. Horizontal and vertical EOG was recorded.

*TMS protocol*: A Mag&More PowerMag Research 100 TMS stimulator (Mag&More®) with a 7 cm figure-eight coil (Mag&More®) was used. In TMS trials a 50 Hz triple-pulse at 80% of individual resting motor threshold was delivered. For experiment 3a the triple-pulse was delivered to the vertex (the stimulation site was EEG electrode site Cz; mean stimulation intensity was 43.0 (SD = 5.5)% maximal stimulator output). Whereas for experiment 3b the triple-pulse was delivered to the right parietal cortex (over EEG electrode position CP6 as identified in experiment 1 and also used as stimulation site in experiment 2; with a mean stimulation intensity of 41.8 (SD = 6.9)% maximal stimulator output). For both experiments the triple-pulse was delivered during the delay period with an onset jittered across trials (see above). EEG was recorded throughout the experiment.

*EEG data analysis*: Preprocessing and artefact rejection was done exactly as described for experiment 2. Data were segmented into epochs starting 1000 ms prior to and ending at onset of the TMS triple-pulse. For electrode site AFz segments were then filtered between 4 and 7 Hz, and instantaneous phase was derived. EEG phase at TMS onset was estimated based on the value gathered 91 ms prior to the triple-pulse (half period of the centre frequency at 5.5 Hz) to avoid results being influenced by filter ringing artefacts at the segment edges. Trials were then sorted into ten FM-theta phase bins of 36˚ and the rate of correct responses was calculated for each of the theta phase bins.

One-way repeated-measures ANOVAs with factor THETA PHASE comparing accuracy rates across the ten FM-theta phase bins was run.

### Experiment 4

*Subjects*: 15 healthy volunteers (14 females; mean age 22.7 years, (SD = 2.4)) were tested for experiment 4 after giving written informed consent. A priori power analysis was carried out with G*Power^47^. Effect size was based on experiment 2. However, since a small sample size was tested in experiment 2 and effect size was very high, only a third of the reported effect size from experiment 2 was used in a priory power analysis. With f = .2, alpha = .05 and 1-beta = .8 a sample size of 14 was suggested. All but three participants were right handed, and none of the volunteers suffered from any psychiatric or neurological disorders. Participants had normal or corrected to normal vision. One participant did not finish the condition with frontal TMS due to a high level of discomfort, and one other participant had to be excluded from statistical analysis because he/she showed very low performance (more than 2.5 std below the mean) in the condition with parietal TMS. The experiment was approved by the LMU Munich F11 ethics committee.

*Experimental design*: A similar experimental design as in experiment 2 was used. However, a within-subject design with two TMS sessions on different days was applied. In one session participants received TMS over right parietal cortex (10-10 system EEG electrode position CP6) exactly the same way as in experiment 2. In the other session TMS was delivered over right frontal cortex (electrode position FC6), which should control better for cutaneous sensation and muscle twitches than stimulation over the vertex like the one in experiment 3a^29,30^. The within-subjects design in this experiment allowed a direct comparison between TMS delivered over the parietal site and a control site. Moreover, the CP6 stimulation session was supposed to act as an internal replication attempt for experiment 2.

*EEG data acquisition*: EEG data were recorded from 30 scalp sites arranged according to the extended 10-10-system using a BrainAmp MRplus amplifier (BrainProducts®) and a TMS compatible electrode cap with Ag-AgCl electrodes (Easycap®). Signal was acquired at a rate of 1000 Hz in a frequency range between 0.016 and 80 Hz (with a notch filter at 50 Hz). During recording a reference on the tip of the nose was used and the ground electrode was placed at electrode position FPz. Horizontal and vertical EOG was recorded.

*TMS protocol*: A Mag&More PowerMag Research 100 TMS stimulator (Mag&More®) with a 7 cm figure-eight coil (Mag&More®) was used. In TMS trials a 50 Hz triple-pulse at 80% of individual resting motor threshold was delivered. For the right parietal TMS session the triple-pulse was delivered at EEG electrode position CP6 (identical to experiment 2; mean stimulation intensity was 40.3 (SD = 4.8)% maximal stimulator output). Whereas for the right frontal TMS session the triple-pulse was delivered at EEG electrode position FC6 (same stimulation intensity as in the other session; however, in six participants the intensity had to be lowered as 80% resting motor threshold was too uncomfortable to bear (mean stimulation intensity was 37.3 (SD = 5.9)% maximal stimulator output); one participant did not finish this session due to a too high level of discomfort). For both sessions the triple-pulse was delivered during the delay period with an onset jittered across trials (see above). TMS coil position was monitored using a frameless stereotactic neuronavigation device (Mag&More® PowerMag View!). EEG was recorded throughout the experiment.

*EEG data analysis*: Preprocessing and artefact rejection was done exactly as described for experiment 2. Data were segmented into epochs starting 1000 ms prior to and ending at onset of the TMS triple-pulse. For electrode site AFz segments were then filtered between 4 and 7 Hz, and instantaneous phase was derived. EEG phase at TMS onset was estimated based on the value gathered 91 ms prior to the triple-pulse (half period of the centre frequency at 5.5 Hz) to avoid results being influenced by filter ringing artefacts at the segment edges. Trials were then sorted into ten FM-theta phase bins of 36˚ and the rate of correct responses was calculated for each of the theta phase bins.

A two-way repeated-measures ANOVA was run on correct response rates with factors TMS SITE (CP6 vs. FC6) and THETA PHASE. To evaluate whether findings from experiments 2 and 3 were replicated/reproduced by experiment 4 two one-way repeated-measures ANOVAs with factor THETA PHASE were run separately for stimulation site CP6 (like in experiment 2) and FC6 (as a control like in experiment 3). Finally, data sets from experiment 2 and the CP6 stimulation condition from experiment 4 were merged to run a one-way repeated-measures ANOVA with factor THETA PHASE on a larger sample.

### Reporting summary

Further information on research design is available in the Nature Research Reporting Summary linked to this article.

## Supplementary information

Supplementary Information

Reporting Summary

## Data Availability

Data will be provided by the corresponding author on Open Science Framework (https://osf.io/p529m/) upon request.

## Code availability

Custom code will be provided by the corresponding author on Open Science Framework (https://osf.io/p529m/) upon request.
