# Supplementary material for: Dynamic regulation of interregional cortical communication by slow brain oscillations during working memory
Source: Nat Commun. 2019 Sep 18;10:4242. doi: 10.1038/s41467-019-12057-0 (PMC6751161; doi:10.1038/s41467-019-12057-0)
Supplement: Supplementary file 1 — Supplementary Information [file 41467_2019_12057_MOESM1_ESM.pdf]

Supplementary Online Material

Dynamic regulation of interregional cortical communication by slow brain oscillations during working memory

Berger, B., Griesmayr, B., Minarik, T., Biel, A. L., Pinal, D., Sterr, A., Sauseng, P.

Supplementary Discussion

Reliability of pre-TMS phase prediction. In experiments 2, 3 and 4 FM-theta phase at TMS onset was estimated by using instantaneous phase 91 ms prior to stimulation and adding half a 5.5 Hz period. To evaluate how reliably FM-theta phase was predicted by this method EEG segments without any TMS artefact were analysed from the CP6 stimulation data set from experiment 4. In each of the trials a virtual marker was set and instantaneous FM-theta phase was obtained for this time point. Then pi/2 was added to instantaneous FM-theta phase from 91 ms before the virtual marker, and this phase value was subtracted from the one obtained at the marker. Thus, if FM-theta phase at the virtual marker was precisely predicted by the method used in experiments 2, 3 and 4 the phase difference was expected to be exactly zero. For each participant phase difference vectors (one for each trial) were then averaged. The length of this mean vector (ranging from 0 to 1) indicates how stable the phase difference across trials was, with a length of 1 meaning that there is no phase variance across trials at all, and 0 indicating that phase differences across trials being completely random. Accordingly, phase prediction with absolute precision and no error at all should result in a mean vector length of 1 and a mean vector direction at pi/-pi. A small mean vector length, in contrast, would suggest high variability of phase difference across single trials, and a mean vector direction deviance from pi/-pi would indicate a systematic bias in phase prediction.

Mean vector length was tested on being significantly larger than chance [S1]. This was confirmed for each single participant ($p<.000001$) suggesting that phase differences were fairly consistent across trials. As can be seen in supplementary figure 1 mean vector direction was within a range of about +/- 30° around pi/-pi across participants. For a FM-theta at 5.5 Hz this would mean that for participants with the biggest deviation there was a temporal imprecision of predicting FM-theta phase of about 15 ms.

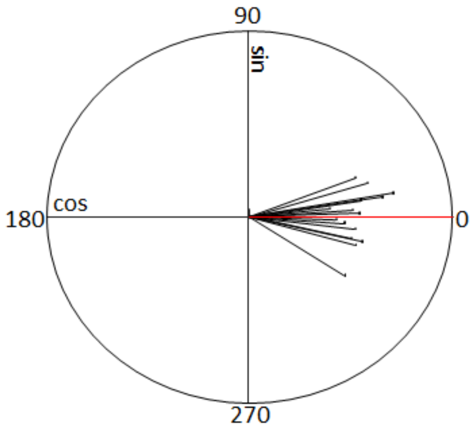

Supplementary Figure 1: Pre-TMS theta phase prediction. Mean vector length of phase differences between predicted and actual FM-theta phase values are plotted for each single participant. Perfectly correct prediction would be indicated by a mean vector length of 1 (i.e. the full radius of the circle) and a mean direction of 0° (see the red line). A mean vector with a random direction and a very short mean length would indicate poor prediction. All the participants showed statistically highly significant mean vector length with a clear tendency towards 0° mean direction. This argues for good prediction of pre-TMS FM-theta phase with low variability across trials.

Supplementary Figures

a

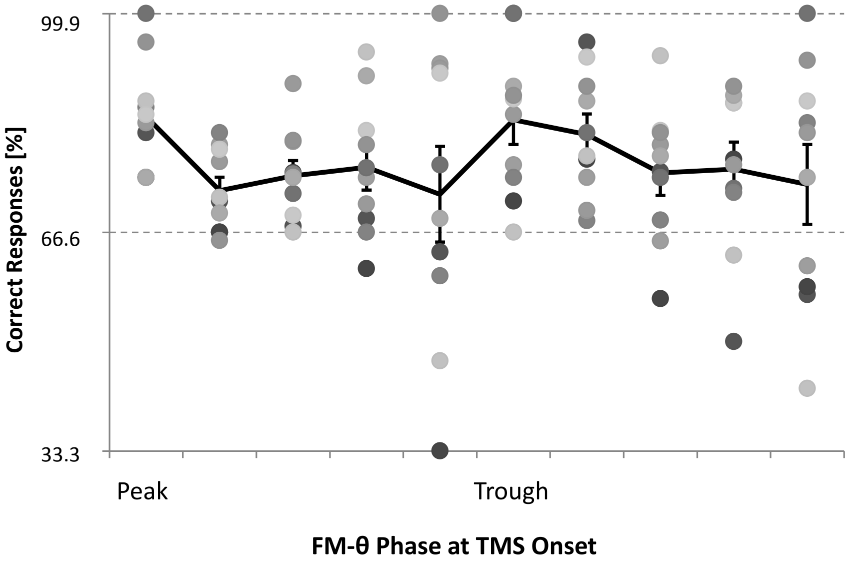

b

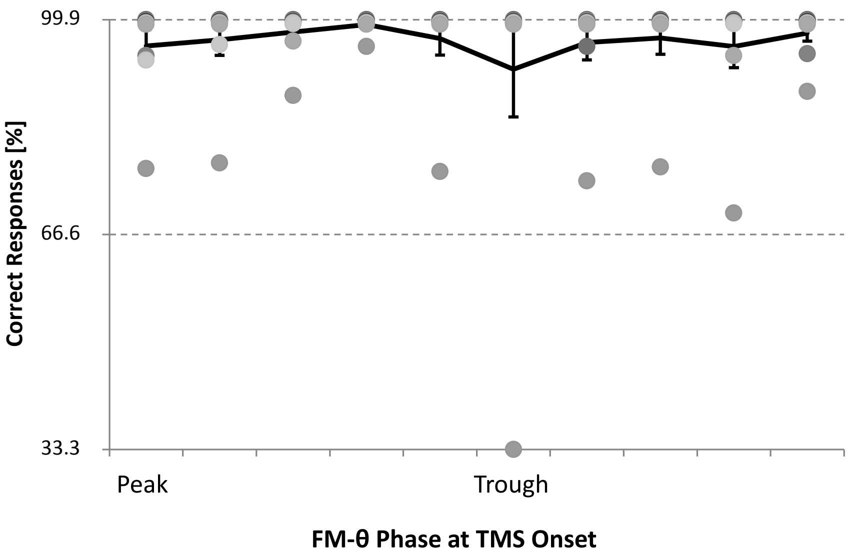

Supplementary Figure 2: Results from experiments 3a and 3b. Percentage of correct responses as a function of FM-theta phase at TMS triple-pulse onset with TMS applied over the vertex (a) while participants were doing the same task as in experiment 2, and

(b) TMS over electrode position CP6 while participants were performing the easiest task condition – retention of only one item. Dots represent single subject data. The line graph represents sample mean values with standard error of mean as error bars. Note that in (b) many participants were performing at 100% correct responses. Therefore, their data points are overlaid and individually invisible.

a

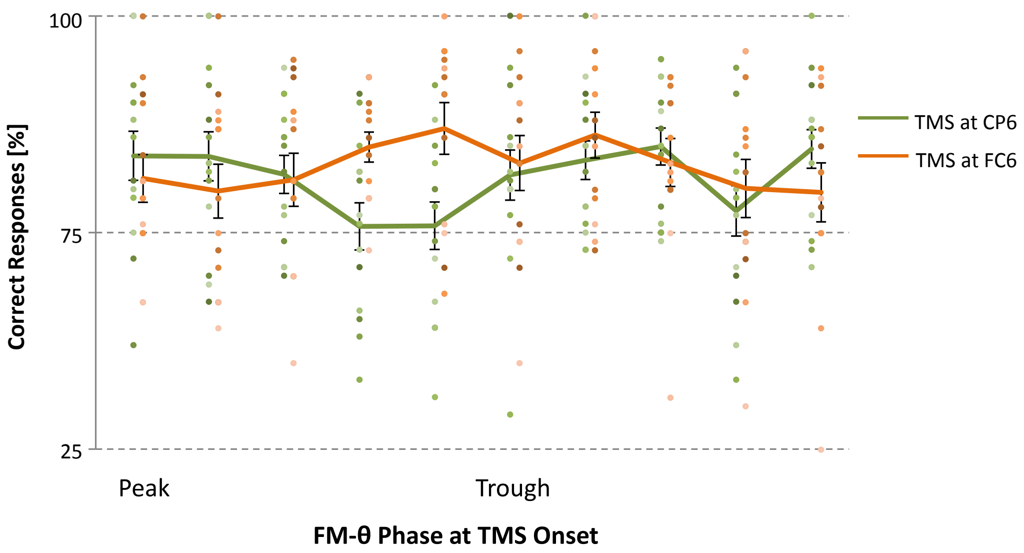

b

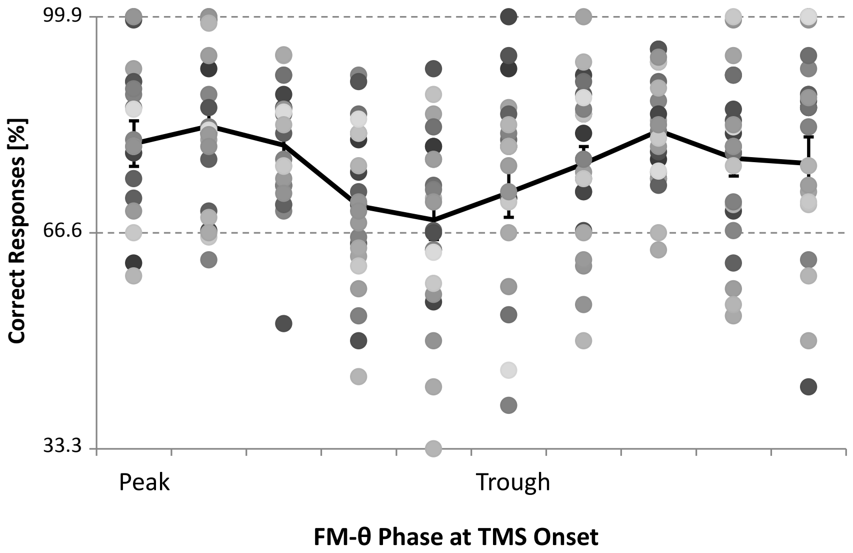

Supplementary Figure 3: Results from experiment 4 and merged data from experiment 2 and 4. A) Percentage of correct responses as a function of FM-theta phase at TMS triple-pulse onset with TMS applied at electrode site CP6 (identical to experiment 2) in green and electrode site FC6 in orange. Dots in green represent individual participants' data for the CP6

stimulation condition, dots in orange data for FC6 TMS condition. The line graphs represent sample mean values with standard error of mean as error bars. Note that in the CP6 condition a similar pattern as in experiment 2 is obtained despite an overall higher performance level of the participants in experiment 4. B) Data from experiment 2 and experiment 4 with TMS over CP6 were merged. Again, dots represent individual participants' data points, and the line graph represents sample mean values with standard error of mean as error bars.

Supplementary Reference

[S1] Thompson, R.O.R.Y. Coherence significance levels. *Journal of the Atmospheric Sciences,* **36**, 2020-2021 (1979).
