# Supplementary material for: Dynamic regulation of interregional cortical communication by slow brain oscillations during working memory
Source: Nat Commun. 2019 Sep 18;10:4242. doi: 10.1038/s41467-019-12057-0 (PMC6751161; doi:10.1038/s41467-019-12057-0)
Supplement: Supplementary file 2 — Reporting Summary [file 41467_2019_12057_MOESM2_ESM.pdf]

natureresearch

Corresponding author(s): Paul Sauseng

Last updated by author(s): Aug 7, 2019

Reporting Summary

Nature Research wishes to improve the reproducibility of the work that we publish. This form provides structure for consistency and transparency in reporting. For further information on Nature Research policies, see Authors & Referees and the Editorial Policy Checklist.

Statistics

For all statistical analyses, confirm that the following items are present in the figure legend, table legend, main text, or Methods section.

n/a	Confirmed	
☐	☒	The exact sample size (*n*) for each experimental group/condition, given as a discrete number and unit of measurement
☐	☒	A statement on whether measurements were taken from distinct samples or whether the same sample was measured repeatedly
☐	☒	The statistical test(s) used AND whether they are one- or two-sided<br>*Only common tests should be described solely by name; describe more complex techniques in the Methods section.*
☒	☐	A description of all covariates tested
☐	☒	A description of any assumptions or corrections, such as tests of normality and adjustment for multiple comparisons
☐	☒	A full description of the statistical parameters including central tendency (e.g. means) or other basic estimates (e.g. regression coefficient) AND variation (e.g. standard deviation) or associated estimates of uncertainty (e.g. confidence intervals)
☐	☒	For null hypothesis testing, the test statistic (e.g. *F*, *t*, *r*) with confidence intervals, effect sizes, degrees of freedom and *P* value noted<br>*Give P values as exact values whenever suitable.*
☐	☒	For Bayesian analysis, information on the choice of priors and Markov chain Monte Carlo settings
☒	☐	For hierarchical and complex designs, identification of the appropriate level for tests and full reporting of outcomes
☐	☒	Estimates of effect sizes (e.g. Cohen's *d*, Pearson's *r*), indicating how they were calculated

Our web collection on statistics for biologists contains articles on many of the points above.

Software and code

Policy information about availability of computer code

Data collection	Neurobehavioural Systems Presentation, BrainVision Recorder, PowerMag View!
Data analysis	BrainVision Analyzer, Matlab, SPSS, JASP

For manuscripts utilizing custom algorithms or software that are central to the research but not yet described in published literature, software must be made available to editors/reviewers. We strongly encourage code deposition in a community repository (e.g. GitHub). See the Nature Research guidelines for submitting code & software for further information.

Data

Policy information about availability of data

All manuscripts must include a data availability statement. This statement should provide the following information, where applicable:

- Accession codes, unique identifiers, or web links for publicly available datasets
- A list of figures that have associated raw data
- A description of any restrictions on data availability

Data Availability Statement
Data will be provided by the corresponding author on Open Science Framework (https://osf.io/p529m/) upon request.

Field-specific reporting

Please select the one below that is the best fit for your research. If you are not sure, read the appropriate sections before making your selection.

☐ Life sciences ☒ Behavioural & social sciences ☐ Ecological, evolutionary & environmental sciences

For a reference copy of the document with all sections, see nature.com/documents/nr-reporting-summary-flat.pdf

Behavioural & social sciences study design

All studies must disclose on these points even when the disclosure is negative.

Study description	quantitative experimental data
Research sample	LMU Munich, University of Surrey and University of Salzburg undergraduates and postgraduates
Sampling strategy	convenience sampling; a priori power analyisis;
Data collection	behavioural data were recorded on a PC viaa button press; for neurophysiological data multi-channel EEG amplifiers were used; TMS was delivered with a commercial stimulator;
Timing	EEG data acquisition for experiment 1 started in 2009.
Data exclusions	in experiment 1, five participants had to be excluded due to a high number of artifacts in the EEG; this is reported in paragraph 1 of the Methods section; in experiments 2 and 4 there was one participant each who performed 2.5 SD below the mean and/or on chance level. These participants were excluded from statistical analysis. The procedure is clearly stated in the methods section (paragraphs on data analysis for experiment 2 and experiment 4).
Non-participation	in experiment 4 one participant did not finish the second stimulation session.
Randomization	n.a. (within subject design)

Reporting for specific materials, systems and methods

We require information from authors about some types of materials, experimental systems and methods used in many studies. Here, indicate whether each material, system or method listed is relevant to your study. If you are not sure if a list item applies to your research, read the appropriate section before selecting a response.

Materials & experimental systems

n/a	Involved in the study	
☒	☐	Antibodies
☒	☐	Eukaryotic cell lines
☒	☐	Palaeontology
☒	☐	Animals and other organisms
☐	☒	Human research participants
☒	☐	Clinical data

Methods

n/a	Involved in the study	
☒	☐	ChIP-seq
☒	☐	Flow cytometry
☒	☐	MRI-based neuroimaging

Human research participants

Policy information about studies involving human research participants

Population characteristics	no covariate-relevant population characteristics.
Recruitment	participants were recruited via flyers at the universities;
Ethics oversight	University of Surrey Ethics Committee and LMU F11 Ethics Review Board

Note that full information on the approval of the study protocol must also be provided in the manuscript.
